# Clinical characteristics and prognosis of patients with incidentally discovered chest wall sarcoma compared with those of symptomatic patients

**DOI:** 10.1093/jjco/hyae059

**Published:** 2024-05-08

**Authors:** Jun Iwatsu, Shinichirou Yoshida, Munenori Watanuki, Shin Hitachi, Sota Oguro, Mika Watanabe, Toshimi Aizawa

**Affiliations:** Department of Orthopedic Surgery, Tohoku University Graduate School of Medicine, Sendai, Japan; Department of Orthopedic Surgery, Tohoku University Graduate School of Medicine, Sendai, Japan; Department of Orthopedic Surgery, JR Sendai Hospital, Sendai, Japan; Department of Diagnostic Radiology^,^, Tohoku University Hospital, Sendai, Japan; Department of Diagnostic Radiology^,^, Tohoku University Hospital, Sendai, Japan; Department of Pathology, Tohoku Kosai Hospital, Sendai, Japan; Department of Orthopedic Surgery, Tohoku University Graduate School of Medicine, Sendai, Japan

**Keywords:** medical checkups, chest wall sarcoma, incidental, cancer screening, asymptomatic

## Abstract

**Objective:**

Sarcomas of the bone and soft tissues are detected after the onset of pain, detectable mass and related symptoms in the absence of a standardized screening examination. However, primary chest wall sarcomas can be incidentally detected upon chest X-ray or computed tomography. Previous studies of incidental primary chest wall sarcomas lack prognosis and disease-specific clinical data. This study aimed to investigate the prognoses of patients with incidental chest wall sarcomas and compare them with those of symptomatic patients.

**Methods:**

This study included 18 patients diagnosed with primary chest wall sarcoma between 2010 and 2023. Patient information such as age, sex, tumour diameter, tumour location, symptoms, treatment, time to treatment initiation, pathological diagnosis and outcome were retrospectively analysed.

**Results:**

Among the 18 patients, the sarcomas were incidentally detected in five by chest X-ray and computed tomography in three and two patients, respectively. The pathological diagnoses of the patients were Ewing sarcoma, Chondrosarcoma grade 1, grade 2, periosteal osteosarcoma and malignant peripheral nerve sheath tumour. The patients had no symptoms at the first visit to our hospital, and no lesions in other organs were detected at the time of the initial examination. At the final follow-up, the patients remained disease-free after radical treatment. The tumour sizes of the five patients were significantly smaller than those of patients with symptoms (*P* = 0.003).

**Conclusions:**

The incidental detection of chest wall sarcomas and consequent early detection and treatment of tumours improves patient prognosis relative to that of symptomatically diagnosed patients.

## Teaser

Incidental detection of chest wall sarcomas improves prognosis in patients with tumours.

## Introduction

Primary chest wall tumours of the bone and soft tissues are uncommon, and their management is challenging due to the broad differential diagnoses, including benign and malignant tumours (1). Further, some primary chest wall chondrosarcomas (CSs) appear normal on the initial radiograph, and this delay in diagnosis is significantly associated with tumour-related death (2). This indicates that early detection and diagnosis are crucial for chest wall sarcomas, as well as for other sarcomas. However, almost all patients with sarcomas are detected after the onset of its symptoms, such as pain or a mass, (3) because there are no screening examinations for sarcomas.

Cancer screening is performed as a public health intervention for the early detection of cancers before any symptoms appear, usually by computed tomography (CT), chest X-ray (CXR) or blood tests. In Japan, CXR is performed as a routine medical checkup at schools and workplaces to detect potential respiratory diseases such as lung cancer and tuberculosis (4). CXR screening is considered more effective for the eventual treatment outcome of lung cancers than no screening (5). Furthermore, chest wall sarcomas are occasionally discovered incidentally during CXR (6–9). Aydogdu et al. (9) reported that four of the 78 cases of primary rib tumours were malignant, asymptomatic and accidental findings. Covello et al. (6) described an incidental Askin tumour of the rib on preoperative radiography during surgery for a thumb fracture. However, the prognoses of these incidentally detected chest wall sarcomas were not specified in either report. Therefore, this study aimed to investigate the prognoses of patients with incidental malignant chest wall sarcomas and compare them with those of symptomatic patients.

## Methods

**Figure 1:**
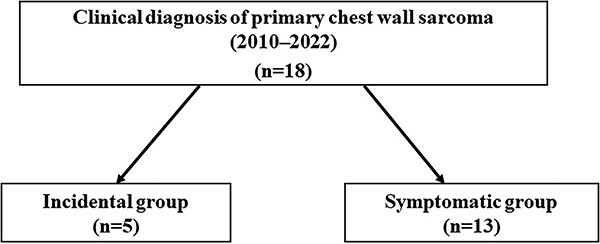
Study flow chart.

This retrospective study was conducted in accordance with the ethical standards of the Declaration of Helsinki and approved by the Institutional Review Board of Tohoku University Hospital (approval number: 2202-1-658). Between 2010 and 2023, 18 patients diagnosed with primary chest wall sarcoma in our department were enrolled in this study upon receiving their written informed consent. Chest wall sarcomas were defined as malignant neoplasms involving the ribs and soft tissues overlying the ribs. The cases were divided into two groups. The incidental group included those in whom the neoplasm was accidentally discovered during medical checkups or examinations for other diseases. The symptomatic group included those who visited a hospital with symptoms by themselves (Fig. 1). Patient information such as age, sex, tumour diameter, tumour site, tumour depth, treatment, time to treatment initiation (TTI), pathological diagnosis and prognosis were retrospectively obtained. The American Joint Committee on Cancer (AJCC) staging system, eighth edition, was used to define tumour grade (10). TTI was defined as the time from the patient’s first visit to our hospital to the initiation of the first definitive treatment. The first definitive treatment not only included surgery but also preoperative chemotherapy and radiotherapy. Overall survival (OS) was defined as the time from the patient’s first visit to our hospital to death from any cause or last follow-up.

For preoperative diagnosis, CT-guided biopsy, core needle biopsy or incisional biopsy was performed by radiologists or orthopedic surgeons. An experienced pathologist diagnosed the specimens. The tumour diameter and time to treatment were analysed using an unpaired t-test. OS was estimated using the Kaplan–Meier method, and the log-rank test was used for univariate analyses to determine the difference in the survival. The data were calculated using SPSS Version 29.0 (SPSS Japan Inc., Tokyo, Japan), and *P* < 0.05 was considered statistically significant.

## Results

**Figure 2:**
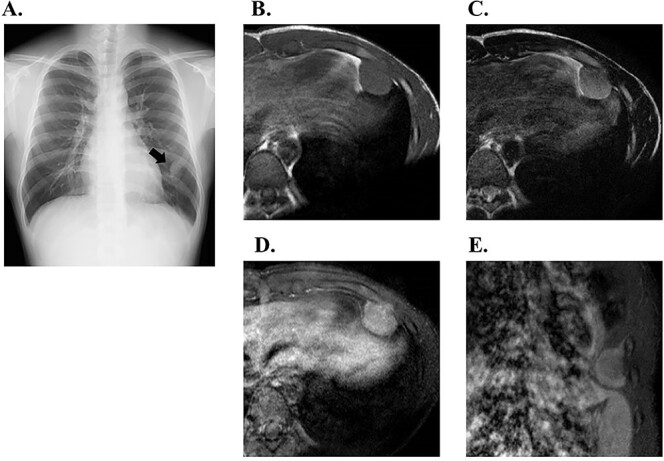
Chest X-ray of an 18-year-old male referred to our hospital (Case 4). A medical checkup in high school revealed the tumour at the left sixth rib (A, black arrow). He had no complaints, and the tumour was not palpable. Magnetic resonance imaging (MRI) revealed a 37 × 34 × 20 mm mass in contact with the pleura and located on the thoracic side. (B–E) He was diagnosed as having Ewing sarcoma by computed tomography-guided biopsy. A total of 29 days after his first visit to our hospital, he started to undergo neoadjuvant chemotherapy, surgery and adjuvant chemotherapy. At the last follow-up, he achieved a continuous disease-free status. Axial view MRI: T1-weighted image (B), T2-weighted image (C), Fat-suppressed contrast-enhanced T1-weighted image (D), coronal view MRI: Fat-suppressed contrast-enhanced T1-weighted image (E).

A total of 18 patients were included in this study. Among them, five patients’ chest wall sarcomas were discovered incidentally: three by CXR during routine medical checkups, one during CT medical checkups for lung cancer and the other by CT for a different disease (Fig. 2). The other 13 patients were diagnosed with chest wall sarcomas during their hospital visits for symptoms such as pain or a mass.

**Figure 3:**
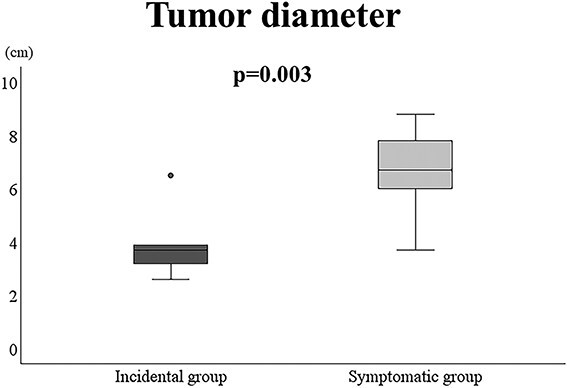
Tumour diameter in the incidental and symptomatic groups. Maximum tumour diameter was measured using MRI. The maximum tumour diameter at the first visit was 4.0 (2.6–6.5) cm in the incidental group and 6.7 (3.7–8.8) cm in the symptomatic group. The tumour diameter in the incidental group was significantly smaller than that in the symptomatic group (*P* = 0.003).

Table 1 shows a summary of the 18 patients included in this study. The median age was 56 and 49 years in the incidental and symptomatic groups, respectively. The median follow-up periods were 48 and 55 months in the incidental and symptomatic groups, respectively. The pathological diagnoses in the incidental group were CS Grade 2 and Grade 1, Ewing’s sarcoma, periosteal osteosarcoma and malignant peripheral nerve sheath tumour in each of the five cases. The remaining 13 cases were CS Grade 2 in four cases, Ewing sarcoma in three, CS Grade 1 in two, and osteosarcoma, epithelioid hemangioendothelioma, alveolar soft part sarcoma and undifferentiated pleomorphic sarcoma in one case each. The AJCC stage in the incidental group with no metastatic lesions was stage IA in three (60%), II in one (20%) and IIA in one (20%) patient, whereas in the symptomatic group, it was IA in three (23%), II in one (8%) and IIA in five (38%) patients. The AJCC stage of two patients with tumours of more than 8 and 5 cm was IIB and IIIA, respectively, and that of two patients with metastatic lesions at the first visit in the symptomatic group was IVA. In all the cases, the incidental group had no symptoms at the first visit. In contrast, the symptomatic group presented with complaints of a mass in five cases, pain in six cases and both in two cases. The maximum tumour diameter at the first visit was 4.0 (2.6–6.5) cm in the incidental group and 6.7 (3.7–8.8) cm in the symptomatic group; the difference was significant (*P* = 0.003) (Fig. 3).

**Table 1:** Summary of the 18 patients in this study

	Case #	Age/sex	Diagnosis	Diameter (cm)	Tumour site	Tumour depth	Metastatic lesion at first visit	AJCC stage	Follow-up duration (months)	Detection method	Symptom
**Incidental** **group**	1	72/F	Chondrosarcoma Grade 1	3.9	Bone	Deep	NO	Stage IA	20	CXR of medical checkup	No
	2	64/M	Chondrosarcoma Grade 2	6.5	Bone	Deep	NO	Stage IIA	40	CXR of medical checkup	No
	3	70/M	Periosteal osteosarcoma	2.6	Bone	Deep	NO	Stage IA	28	CT for other disease	No
	4	18/M	Ewing sarcoma	3.7	Soft tissue	Deep	NO	Stage II	100	CXR of medical checkup	No
	5	55/F	Malignant peripheral nerve sheath tumour	3.2	Soft tissue	Deep	NO	Stage IA	52	CT of medical checkup	No
**Symptomatic** **group**	6	17/F	Ewing sarcoma	8.3	Bone	Deep	NO	Stage IIB	128	Symptom	Pain
	7	54/M	Chondrosarcoma Grade 2	5.8	Bone	Deep	NO	Stage IIA	112	Symptom	Mass
	8	39/M	Chondrosarcoma Grade 1	6.7	Bone	Deep	NO	Stage IA	100	Symptom	Mass
	9	75/M	Conventional Osteosarcoma	7.8	Bone	Deep	YES	Stage IVA	27	Symptom	Pain
	10	34/M	Ewing sarcoma	7.1	Bone	Deep	NO	Stage IIA	51	Symptom	Pain
	11	58/M	Epithelioid hemangioendothelioma	6.3	Bone	Deep	NO	Stage IA	77	Symptom	Pain
	12	62/M	Chondrosarcoma Grade 2	6.0	Bone	Deep	NO	Stage IIA	41	Symptom	Mass
	13	72/F	Chondrosarcoma Grade 1	3.7	Bone	Deep	NO	Stage IA	20	Symptom	Pain
	14	34/M	Chondrosarcoma Grade 2	7.2	Bone	Deep	NO	Stage IIA	11	Symptom	Pain
	15	44/F	Ewing sarcoma	6.3	Bone	Deep	NO	Stage IIA	10	Symptom	Mass and pain
	16	73/M	Chondrosarcoma Grade 2	8.8	Bone	Deep	YES	Stage IVA	11	Symptom	Mass and pain
	17	20/M	Alveolar soft part sarcoma	8.0	Soft tissue	Deep	NO	Stage IIIA	48	Symptom	Mass
	18	53/F	Undifferentiated pleomorphic sarcoma	4.7	Soft tissue	Deep	NO	Stage II	78	Symptom	Mass

M = male; F = female; CT = computed tomography; CXR = chest X-ray

**Figure 4:**
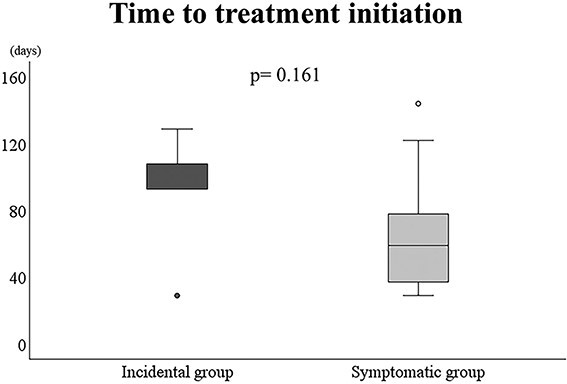
Time to treatment initiation (TTI) from the first visit in the incidental and symptomatic groups. TTI was defined as the time from the patient’s first visit to our hospital to the initiation of the first definitive treatment. The average TTI was 93 days (29–129) in the incidental group and 65 days (29–144) in the symptomatic group (*P* = 0.161). TTI from the first visit was not significantly different between the two groups (*P* = 0.161).

**Figure 5:**
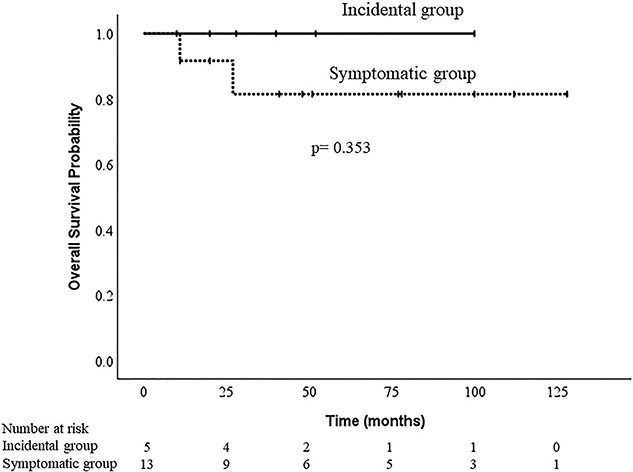
The Kaplan–Meier curve illustrates the overall survival between incidental and symptomatic groups.

Table 2 shows the time to treatment, treatment type and outcomes. The average TTI from the first visit was 73 days; this did not significantly differ between the two groups: incidental group, 93 days (29–129), symptomatic group, 65 days (29–144) (*P* = 0.161) (Fig. 4). The incidental group underwent radical treatment for each tumour. In contrast, 11 patients in the symptomatic group underwent radical treatment, and two cases (cases 9 and 16) with metastasis at the first visit to our hospital underwent palliative chemotherapy. In terms of patient outcomes in the incidental group, there were no other organ lesions at the time of the initial examination and no recurrence or metastasis during follow-up. In contrast, in the symptomatic group, three cases (cases 10, 12 and 17) showed lung metastasis during follow-up despite no delay in treatment (mean TTI in three cases was 46 days). Two patients (patients 9 and 16) died of the disease (Table 3). The Kaplan-–Meier curve showed that the 1-year OS was 100% and 91.7%, and the 3-year OS was 100% and 81.5% in the incidental and symptomatic groups, respectively (log-rank, *P* = 0.353) (Fig. 5).

**Table 2:** Clinical details of the 18 patients in this study

	Case #	TTI from first visit (days)	Surgery	Radiotherapy	Chemotherapy	Outcome
Incidental group	1	129	YES	NO	NO	CDF
	2	93	YES	NO	NO	CDF
	3	108	YES	NO	NO	CDF
	4	29	YES	NO	YES	CDF
	5	108	YES	NO	NO	CDF
Symptomatic group	6	29	YES	NO	YES	CDF
	7	78	YES	NO	NO	CDF
	8	64	YES	NO	NO	CDF
	9	29	NO	YES	YES	DOD
	10	42	YES	YES for metastatic lesion	YES	AWD
	11	122	YES	NO	NO	CDF
	12	59	YES	YES for metastatic lesion	NO	AWD
	13	37	YES	NO	NO	CDF
	14	93	YES	NO	NO	CDF
	15	64	YES	YES	YES	NED
	16	144	NO	NO	YES	DOD
	17	36	YES	NO	YES	AWD
	18	51	YES	NO	YES	CDF

TTI; Time to treatment initiation; CDF: continuous disease-free; NED: No evidence of disease; AWD: alive with disease; DOD: dead of disease

**Table 3:** Status at last follow-up

	Incidental group	Symptomatic group
CDF	5	8
NED	0	0
AWD	0	3
DOD	0	2

CDF: continuous disease-free; NED: no evidence of disease; AWD: alive with disease; DOD: dead of disease

## Discussion

This study is the first to compare patients with incidentally discovered chest wall sarcomas and symptomatic patients. This study revealed that the tumour diameter in the incidental group was significantly smaller than that in the symptomatic group. In addition, the prognosis of the incidental group was favourable.

Almost all patients with sarcomas visit the hospital after the onset of symptoms such as a mass and pain due to the lack of screening examinations for sarcomas. Therefore, the time from symptom onset to initiation of treatment should be shortened. Some reports have emphasized that early diagnosis is necessary to improve patient prognosis (11–13). Nakamura et al. (12) reported that a longer period from symptom onset to diagnosis or initial treatment influenced patient survival. Ogura et al. (13) recommended that patients with high-grade soft tissue sarcomas should undergo treatment within 30 days of diagnosis. Based on these results, early detection of the tumour and initiation of treatment are found to be crucial for good patient outcomes. In this study, three patients who underwent radical treatment as early as possible (mean TTI, 46 days) showed lung metastasis during follow-up. This may be due to the significant delay before the first visit to the hospital. The TTI from the first visit was not significantly different between the two groups, indicating that the difference in the time to the first visit to our hospital between the two groups caused a difference in prognosis.

To detect tumours as early as possible, routine screening for breast and lung cancers is routinely performed (14,15), and these tests are reportedly effective in detecting other types of cancers (16). Additionally, annual screening for lung cancer with low-dose chest CT is recommended (15) so that incidental chest wall sarcomas can be detected at the same time (17). In this study, five cases were discovered incidentally: three on CXR and two on CT.

This study emphasizes the necessity of checking extrapulmonary areas on CXR or CT. Checking for lesions that are included in the image and responding appropriately if found may affect patient prognosis. In addition, 18F-fluorodeoxyglucose-positron emission tomography (FDG-PET) was used as a cancer screening tool in Japan, and five sarcomas were detected using a screening programme conducted between 2006 and 2009 (18). FDG-PET may also be useful for early detection of deep asymptomatic soft tissue and bone sarcomas.

Some reports have described an association between tumour size and prognosis or local recurrence (19–21). Alvarado et al. (20) reported that larger tumour size was significantly associated with OS in patients with chest wall CS. A smaller tumour diameter indicates that the tumours were discovered earlier than when they would arise and detectably appear (22). Based on this fact, a smaller tumour diameter in the incidental group proved that the incidental group’s tumours were discovered earlier than in the symptomatic group. The incidental group had no symptoms at the first visit to our hospital, which indicated that the incidental group could be detected with a tumour earlier before the onset of the symptoms than the symptomatic group, following complaints of pain or mass at the first visit to our hospital. Patients with soft tissue sarcoma present with painless and palpable masses (23). In contrast, patients with bone sarcoma tend to present with pain due to infiltration and bone invasion (24). In addition, high-grade bone sarcomas generally result in more pain than that by low-grade bone sarcomas due to early bone invasion. In this study, the proportion of soft tissue sarcoma was higher in the incidental group than in the symptomatic group, and that of low-grade sarcomas (stage IA) was higher in the incidental group than in the symptomatic group.

This study had some limitations. First, as a retrospective study with a small sample size, the findings cannot be extended to diverse patient populations. Secondly, various histological types were included in this study. The prognosis differed according to the histological type. The features of the AJCC stages differed between bone and soft tissue sarcomas; therefore, it is unclear whether tumour size can be used as the standard for tumour progression. Lastly, not all patients completed follow-up. In terms of patient outcomes, the possibility of metastasis or local recurrence occurring in the future, even in the incidental group, cannot be ruled out.

## Conclusion

Chest wall sarcomas can be discovered incidentally on CXR or chest CT. These incidental findings should consider the possibility of a malignant tumour despite being asymptomatic. In this study, the incidental detection of chest wall sarcomas was favourable for early treatment of tumours before the onset of symptoms, improving prognosis.

## Data Availability

*The data underlying this article will be shared on reasonable request to the corresponding author.*
